# Oral Health Status of Illicit Drug Users in a Health District in South Africa

**DOI:** 10.1055/s-0042-1750770

**Published:** 2022-08-01

**Authors:** Ntsakisi Mukhari-Baloyi, Ahmed Bhayat, Thomas K. Madiba, Ntombizodwa R. Nkambule

**Affiliations:** 1 Department of Community Dentistry, Faculty of Health Sciences, University of Pretoria, South Africa

**Keywords:** recreational drugs, oral health, epidemiology, illicit drugs-adverse effects

## Abstract

**Objectives** The prevalence of illicit drug use and its associated oral health complications have been increasing in South Africa (SA). There has been a paucity of studies to determine the oral health status among illicit drug users in SA. This study aimed to determine the oral health knowledge, practices, and status of illicit drug users at rehabilitation centers in a district in SA.

**Materials and Methods** This was a cross-sectional study conducted at four rehabilitation centers in Pretoria, SA. Data were collected using a validated self-administered questionnaire and an oral examination. The questionnaire consisted of three parts: demographics, oral health knowledge, and oral health practices. The oral examination was conducted by a calibrated researcher and included caries, periodontal status, dental erosion, trauma, and soft tissue lesions.

**Statistical Analysis** The data were analyzed using the Statistical Package for Social Sciences (SPSS) software.

**Results** The mean age was 25.5 (±7.49) years with 91% being male. The majority (84%) of patients were Black, and almost all (87%) had never received oral health education; 55% were not aware that illicit drugs could have an impact on the oral health status and 40% had never had a prior dental consultation. The caries prevalence was 68% with a mean decayed, missing and filled teeth (DMFT) score of 5.3 (±6.18). More than half (58%) required periodontal treatment and there were minimal soft and hard tissue lesions diagnosed. Those who perceived their teeth to be in a poor condition had a significantly higher Decayed, Missing and Filled Teeth (DMFT) score compared with those who perceived their oral health to be good. Just under half (41.9%) of the participants did not need any periodontal intervention, with the other half presenting with plaque retention or calculus and thus needed oral hygiene intervention inclusive of scaling and polishing. There was minimal evidence of pathological oral lesions with only 1.5% presenting with necrotizing periodontal disease (NPD). Overall, there was generalized poor oral hygiene

**Conclusion** Many of the participants had poor oral hygiene knowledge and practices and a relatively high prevalence of caries. It is imperative that oral health promotion and education is incorporated into the medical interventions provided at rehabilitation centers.

## Introduction

Illicit drug use, is a global public health problem laden with social and legal implications that inevitably contribute to morbidity and premature mortality. 1 Globally, the risk of exposure to illicit drugs has been reported to have increased by more than 30.2% from 1990 to 2015. 2 In 2020, studies estimated that between 149 and 272 million people aged 15 to 64 years reported to have used a form of illicit drug at least once in that year, and there were over 183,000 drug-related deaths recorded. 3 4

The prevalence of illicit drug use and its health complications has been increasing in South Africa (SA) due to many factors including the porosity of the borders to illegal immigrants and drugs, unstable family structures, physical and emotional abuse, poor socioeconomic status, unemployment, abuse, and a lack of education. 5 6 These illicit drugs include cannabis, cocaine, heroin, methamphetamine (“crystal meth” or “tik”), rock and Mandrax. In September 2018, the South African Constitutional Court ruled that the personal use of cannabis was to be legalized, it was to be made a law within 24 months from the date of the ruling. 7 Due to the timeframe, articles published prior to that will have cannabis listed as an illicit drug.

The oral and dental complications of drug abuse may be related directly to the substance or the way the drugs are consumed. The consumption of drugs could either be oral, intravenous, or by inhalation and the adverse oral health complications that arise from drug use result from direct exposure of the oral tissues to the drugs. The effects on brain function result in a spectrum of addictive behaviors such as risk-taking behavior, poor hygiene, aggression, and carelessness. 8 The use of drugs may lead to alterations in mood, cognition, and thought which affects changes in the user's behavior and attitude toward the hygiene of their teeth. 9 10 Heroin users besides presenting with an altered mental state have been reported as having a negative attitude toward oral health and thus are more likely to have dental anxiety and fear. 9 The users of nyaope, a local combination drug, common in SA, tend to have poor oral hygiene interest, they are known to neglect brushing their teeth completely or only brush on occasion. 11 Although these complications include the oral cavity, many drug users are not aware of the relationship between illicit drug use and oral health conditions.

There has been a paucity of studies to determine the knowledge, practices, and oral health status of illicit drug users in SA. This study aims to determine the oral health status and establish an in depth understanding of the oral health knowledge, attitude, and practices of illicit drug users.

## Materials and Methods

This was an analytical cross-sectional study design. The population included patients admitted for illicit drug addiction at rehabilitation centers within the Tshwane Health District in Pretoria of SA. A list of the drug rehabilitation centers in this district was obtained from by the South African Community Epidemiology Network on Drug Use (SACENDU). There were four registered drug rehabilitation centers listed, and all of them were included in the study. These included the South African National Council on Alcoholism (SANCA) Castle Carey, SANCA Thusong, Stabiliz, and Dr Fabian and Florence Riberio Rehab centers. Although these rehabilitation centers also treat patients with alcoholic dependence issues, only patients who attended for illicit drug use were included in the current study.

A minimum sample size of 190 was required and this was calculated using the 4-year (2015–2018) biannual admission records from the selected rehabilitation centres. 12 The average cumulative total number of inpatients across the chosen centers over the cumulative average of 6 months in the period of 2015 to 2018 was 814. Using the cumulative total number as the population, the data was entered into the Raosoft (2004) online sample size calculator for calculation. The margin of error was set at 5%, confidence level at 95%, population at 814, and response distribution at 80%.

Ethical clearance was obtained for the study, (identifier: 394/2020). The medium of instruction was English and all participants could read and write the language. All participants were given an information leaflet and consent form to read and sign prior to participating in the study. Data were collected by the use of two tools. An English-validated self-administered structured questionnaire and a comprehensive oral examination conducted by the principal researcher. 13

The questionnaire had 18 closed- and open-ended questions that included demographics, knowledge, attitudes, and practices. After completion of the questionnaire, the oral examination was conducted by the researcher who was blinded to the results of the questionnaire. This was done to avoid introduction of bias when completing the oral examination.

The oral examination was done in the rehabilitation centers using a chair and natural light according to the World Health Organization (WHO) guidelines. 14

Dental caries was recorded using the decayed, missing and filled teeth (DMFT) index. The consequences of untreated dental caries was measured using the pulpal involvement (P), ulceration caused by dislocated tooth fragments (U), fistula (F), and abscess (A; PUFA) index. The periodontal status was recorded using the Clinical Community Periodontal Index of Treatment Need (CPITN) index. Trauma and oral lesions were examined and diagnosed by clinical presentation.

An oral health education and promotion seminar was given to all those in the center after every session, in the form of a PowerPoint presentation and demonstrations on models. Those patients who did not want to be included in the study were also invited to attend the presentation.

To ensure intraexaminer reliability, the principal researcher was calibrated for indices and oral lesions using mounted teeth, slides, and images. The Kappa scores obtained ranged from 0.85 to 0.90, and these were considered acceptable. During data collection, every 10th participant was reexamined and results compared with initial results. The kappa scores from the interexaminer reliability ranged between 0.90 and 0.99.

The data collected from the questionnaire and oral examination was entered into Microsoft Office Excel Spreadsheets after which it was checked for completeness, duplicates, and for missing values. Once verified to be complete, the data were exported to the Statistical Package for Social Science Software (SPSS) version 27.0 for statistical analysis. Descriptive and inferential statistics were used to achieve the study objectives. Statistical significance was set at *p* ≤ 0.05. Data were combined from all the centers due to the homogeneity of the participants and the small numbers in each of the four facilities. However, individual results from each center were offered to the respective center managers.

## Results

The mean age of the participants was 25.5 years (± 7.49) with 91% being male and 9% female. The majority (84%) were Black and 94% were from Gauteng. Almost all (87%) had never received oral health education; 55% were not aware that illicit drugs could have a negative impact on the oral health status and 40% had never had a prior dental consultation.

**Fig. 1:**
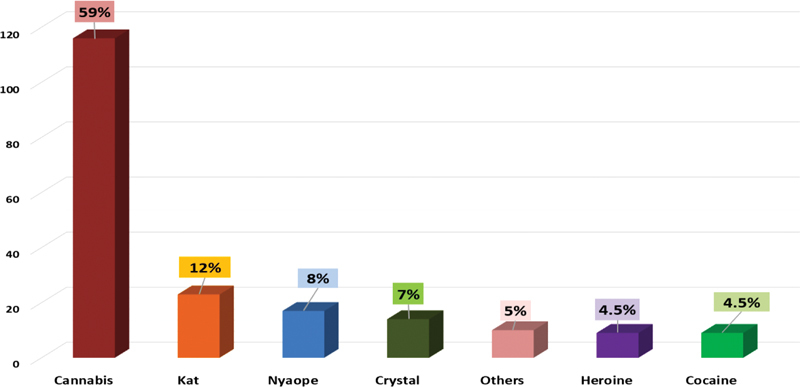
Prevalence of drugs being consumed by participants.

The most commonly used drugs are represented below in Fig. 1 . Cannabis was the most frequently used drug (59%) followed by kat and nyaope.

The most common mode of drug use was smoking (89%), followed by snorting (35%), injection (22%), and ingestion (8%). Regarding the frequency of use, 77% reported to use drugs more than once a week, 13% reported using them several times per week, and 6% at least once per day.

The caries prevalence was 68% with a mean DMFT of 5.3 (± 6.18) as shown in Table 1 . The highest component of the DMFT score was the decayed component (57%) followed by the missing component (32%).

**Table 1:** The mean total and component DMFT scores of the participants ( *n* = 198)

	Decayed	Missing	Filled	Total
Mean ± SD (%)	3.0 ± 3.74 (57%)	1.7 ± 3.71 (32%)	0.6 ± 1.72 (11%)	5.3 ± 6.18 (100)

Abbreviation: SD, standard deviation.

The relationship between the mean DMFT scores and the primary drugs being consumed is shown in Table 2 . The highest mean DMFT score of 10 occurred on a single individual who used “rock” as a primary drug ( Table 2 ). The PUFA prevalence was 2.5%.

**Table 2:** Relationship between the mean DMFT scores and the primary drug being consumed ( *n* = 198)

DMFT	Rock	Benzodiazepine	Codeine	Crystal	Heroine	Ecstasy	Cocaine	Mandrax	Kat	Nyaope	Cannabis	Chemical	Total
Mean	10.00	9.00	9.00	8.21	7.78	6.50	6.22	6.00	5.96	3.53	2.64	0.00	5.28
(± SD)	–	4.43	–	8.54	6.36	7.78	4.38	–	5.75	2.10	4.14	0.00	6.18
Frequency	1	2	1	14	9	2	9	1	23	17	116	3	198

The relationship between the perceived self-reported oral health status and the mean DMFT scores are shown in Table 3 . Those who perceived their teeth to be in a poor condition had a significantly higher mean DMFT score compared with those who had a more positive perception of their oral status.

**Table 3:** Association between self-reported oral health status and mean DMFT scores ( *n* = 198)

Self-reported oral health status	Frequency (%)	Mean (± SD)	*p* -Value
Good	53 (26.8)	3.9 (± 4.19)	<0.001
Average	43 (21.7)	3.2 (± 3.48)	
Poor	86 (43.4)	7.4 (± 7.71)	
I don't know	16 (8.1)	4.0 (± 4.71)	
Total	198	5.3 (± 6.18)	

Abbreviation: SD, standard deviation.

Participants who claimed to brush their teeth occasionally (5%) presented with a significantly higher ( *p* = 0.004) mean total decay score of 8.5 (± 8.11) compared with those who reported to brush twice a day 4.0 (± 4.80; Table 4 ).

**Table 4:** Association of brushing routine and mean decay component ( *n* = 198)

Brushing frequency	Frequency (%)	Mean (± SD)	*p* -Value
Occasionally	10 (5)	8.5 (± 8.11)	0.004
Once a day	111 (56)	3.4 (± 4.51)	
Twice a day	67 (34)	4.0 (± 4.80)	
More than twice daily	10 (5)	7.2 (± 7.54)	
Total	198 (100)	5.3 (± 6.18)	

Abbreviation: SD, standard deviation.

Almost half (43%) had calculus while 42% had a healthy periodontium; only 4% had pockets more than 6-mm deep.

Participants who perceived their gums as poor had a significantly ( *p* = 0.017) higher CPITN score (1.51), compared with those who perceived their gum status as good (1.16; Table 5 ).

**Table 5:** Self-reported perception of gingival status and CPITN scores ( *n* = 198)

	Frequency (%)	Mean (± SD)	*p* -Value
Good	37 (18.7)	1.16 (± 1.04)	0.017
Average	82 (41.4)	1.28 (± 1.18)	
Poor	59 (29.8)	1.51 (± 1.25)	
I don't know	20 (10.1)	0.55 (± 0.99)	
Total	198	1.25 (± 1.18)	

Abbreviations: CPITN, Community Periodontal Index of Treatment Need; SD, standard deviation.

There Only 1.5% presented with necrotizing ulcerative gingivitis and 7.1% presented with lesions such as herpes, bilateral linear alba, heavily coated tongue, aspirin burn, or pericoronitis.

## Discussion

The participant's ages ranged from 18 to 59 years, with a mean age of 25.52 years. The mean age was found to be similar to other studies which reported a mean age between 26 and 29 years. 11 15

It is evident that the majority of drug users are young adults. The age range between 16 and 26 years are considered as the critical decade in an individual's life, it is during these years that a person goes through many phases in attempt to discover themselves. 16 Therefore they are likely to face social and psychological stressors that may predispose them to experimenting or consciously initiating use of illicit drugs.

The majority (91.4%) of drug users were male which was similar to other studies. 11 15 Males seem to be at a higher risk of abusing illicit drugs than females due to societal pressures that may become overwhelming and lead to substance abuse. 17 The societal pressures that men are expected to fulfil are those of the breadwinner, performance of jobs that require strenuous physical labor, and the ability to be able to handle all forms of negative emotional stress well. 18 These reasons, coupled with the lack of seeking professional, help to cope males to be more likely to turn to substance abuse. 19

In SA, illicit drug use affects mostly the Black population as opposed to any other race and this could be due to the fact that 81% of the population is Black. 11 12 In addition, drug use is associated with social factors, such as unemployment and unstable family environments among many other factors, and since the majority of the population is Black, this was not surprising. 6 13

A large number of the participants were not aware that drugs could damage their teeth and this could have been due to the fact that they did not receive any oral health information from the rehabilitation centers. These centers had a daily programmer which included general health education with a nurse, consultation with a medical officer, and sessions with a social worker or psychologist. Although these centers provided general health education, psychological therapy, and support, they did not provide any oral health education. A holistic approach to drug rehabilitation should include oral health care. A healthy mouth also improves esthetics and can have a positive impact on an individual's health, being socially acceptable, and an individuals' self-esteem. 20 21

The relatively high mean DMFT score of 5.8 and the higher decayed component (57%) are common findings in similar studies. This may be attributed to poor oral hygiene routine practices and or a high sugar diet. The mean missing component of the DMFT score contributed 32%, and this may be subject to recall bias as participants could have forgotten why certain teeth were extracted, whether it was due to decay or to trauma. The low F component could be due financial barriers in relation to access to dental care and or delayed treatment seeking for carious teeth.

The acceptable mean adult DMFT score by the WHO is 4. 22 23 Hence it seems that these drug users had a slightly higher DMFT compared with the general population. There are a myriad of factors that may contribute high DMFT in drug users, the chemical composition of the drug(s) used, the salivary pH changes it caused, and/or the psychological effects it might have on the user.

The low PUFA prevalence of 2.5% may be attributed to extractions done due to complications of decay. Untreated caries is often due to barriers to oral health care such as access and cost. 24 25 However, when untreated caries progresses and causes complications, the affected individual is forced to make means to go for treatment, at which stage, the likely possible treatment is extraction. The second molars were the most decayed teeth across all quadrants that may be caused by the presence of an impacted third molar, and/or difficulty to access with cleaning aids. 25 Due to polydrug use, it was difficult to ascertain a direct relation between a single drug and DMFT scores.

Just under half (43%) of the participants had a realistic perception of the state of their teeth in relation to their mean DMFT score. Whereas 18.7% rated their gums as good but were also in need of periodontal intervention. This may be indicative of the compartmentalization between oral health and general health; individuals separate their teeth from the rest of their body when determining their health status. 26 27 They may consider their teeth to be a completely isolated entity, thus it is possible that one can have bad teeth but also have good gums. They see the visual and physical evidence of bad teeth, discoloration, and pain but do not see the difference between healthy gums and diseased gums, hence it may be difficult to refer to the two entities as part of one unit. The other possible explanations could be that poor gum health to a person with low oral health intelligence quotient is associated with bleeding, if there is no bleeding then gums are healthy. 13

A little over half (56.1%) reported to brushing their teeth only once a day. There was a significant difference ( *p* = 0.004) in mean DMFT scores among users in correlation to their brushing routine. Those who reported to brush occasionally had a mean DMFT of 8.5 (± 8.11) compared with those who claimed to brush more than twice a day 7.2 (± 7.54). This was surprising, as it was assumed that those who brush more frequently, would have a much lower mean DMFT score and an improved oral health status. 28 29 This could be due to various reasons including participants claiming to brush their teeth more often than they did as they knew an increased frequency was better for their oral health (response bias), it could be that their behavior changed recently and they increased the frequency of brushing but their teeth had already been damaged in the past hence they recorded a higher mean DMFT score, or that they used to brush more frequently in the past and have since reduced their frequency and this had resulted in their high mean DMFT scores.

Although studies have reported an increased prevalence of oral lesions, such as candidiasis and leukoplakia, with drug use; in the current study, there were very few oral lesions detected. 30 The failure to observe oral lesions in this study may be attributed to the fact that all lesions were diagnosed by visual examination based on distinguishing clinic features only. No specimens were taken for diagnostics tests.

The oral health education package that was used in this study can be used as a temporary measure until research is done to test the effectiveness and impact it can have on the oral health status, knowledge, and practices of illicit drug users. Assuming that this will ensure that health care workers at rehabilitation centers are then able to identify lesions that warrant concern and be able to seek appropriate attention.

## Limitations

Since it was a cross-sectional study design, drug association and presence of oral conditions cannot be confirmed as causal in nature. The missing component of the DMFT score may be subjected to recall bias, and participants could have forgotten if their teeth were extracted due to caries or trauma.

## Conclusion

Many of the participants had poor oral hygiene knowledge and practices and a relatively high prevalence of caries. It is imperative that oral health promotion and education is incorporated into the medical interventions provided at rehabilitation centers.

It is recommended that the health workers at these centers receive training from an oral health professional on oral health screenings and the appropriate referral procedures. This could ensure appropriate referrals where necessary, adequate treatment, and improvement of oral health status of patients.

## References

[1] Lin C Wong B Y Lo M T Chiu Y C Lin Y H Development of an addiction index and delineation 15-year trends of illicit drugs from the Taiwan national drug enhancement database J Psychiatr Res 2020 120 131 136 31670261 10.1016/j.jpsychires.2019.10.016

[2] GBD 2015 Risk Factors Collaborators Forouzanfar M H Afshin A Alexander L T Global, regional, and national comparative risk assessment of 79 behavioural, environmental and occupational, and metabolic risks or clusters of risks, 1990-2015: a systematic analysis for the Global Burden of Disease Study 2015 Lancet 2016 388(10053):1659 1724 27733284 10.1016/S0140-6736(16)31679-8 PMC5388856

[3] Chen C Y Lin K M Health consequences of illegal drug use Curr Opin Psychiatry 2009 22 03 287 292 19378381 10.1097/yco.0b013e32832a2349

[4] Liebenberg J Du Toit-Prinsloo L Steenkamp V Saayman G Fatalities involving illicit drug use in Pretoria, South Africa, for the period 2003 - 2012 S Afr Med J 2016 106 10 1051 1055 27725028 10.7196/SAMJ.2016.v106i10.11105

[5] Begun A L Psychological models of addictive behavior New York Taylor and Francis 2020 95 109

[6] Schotte S Zizzamia R Leibbrandt M Social stratification, life chances and vulnerability to poverty in South AfricaAccessed May 9, 2022 at:https://www.opensaldru.uct.ac.za/bitstream/handle/11090/883/2017_208_Saldruwp.pdf?sequence=1

[7] New cannabis rules proposed for South Africa – to be introduced within next 2 yearsAccessed June 22, 2021 at:https://businesstech.co.za/news/lifestyle/482625/new-cannabis-rules-proposed-for-south-africa-to-be-introduced-within-next-2-years/

[8] Shekarchizadeh H Khami M R Mohebbi S Z Ekhtiari H Virtanen J I Oral health of drug abusers: a review of health effects and care Iran J Public Health 2013 42 09 929 940 26060654 PMC4453891

[9] Abed H Hassona Y Oral healthcare management in heroin and methadone users Br Dent J 2019 226 08 563 567 31028320 10.1038/s41415-019-0206-x

[10] Groenewald C Bhana A Substance abuse and the family: an examination of the South African policy context Drugs Educ Prev Policy 2018 25 02 148 155

[11] Tetarwal A Yengopal V Munshi I Meel R Oral health status among nyaope users at drug rehabilitation clinics in Johannesburg S Afr Dent J 2019 74 01 13 18

[12] Mokwena K “Consider our plight”: a cry for help from nyaope users Health SA Gesondheid 2016 21 137 142

[13] Francis D Webster E Poverty and inequality in South Africa: Critical reflections Dev South Afr 2019 36 06 788 802

[14] Organization WH. Oral health surveys: basic methods-5th editionAccessed May 9, 2022 at:http://www.icd.org/content/publications/WHO-Oral-Health-Surveys-Basic-Methods-5th-Edition-2013.pdf

[15] Harker N Lucas W C Laubscher R Dada S Myers B Parry C D Is South Africa being spared the global opioid crisis? A review of trends in drug treatment demand for heroin, nyaope and codeine-related medicines in South Africa (2012–2017) Int J Drug Policy 2020 83 102839 32650228 10.1016/j.drugpo.2020.102839

[16] Dobson J Life on the Edge: The Next Generation's Guide to a Meaningful Future Carol Stream: IL Tyndale House Publishers, Inc. 2010

[17] Nolen-Hoeksema S Gender differences in risk factors and consequences for alcohol use and problems Clin Psychol Rev 2004 24 08 981 1010 15533281 10.1016/j.cpr.2004.08.003

[18] El-Sawy H Abdel Hay M Badawy A Gender differences in risks and pattern of drug abuse in egypt Egypt J Neurol Psychiat Neurosurg 2010 47 01 413 418

[19] Addis M E Gender and depression in men Clin Psychol Sci Pract 2008 15 03 153

[20] Valencia M LC Peters B Kim N The relationship between income generation, increasing substance dependence and the risk of relapse: a cross-sectional study of drug treatment facilities J Subst Use 2021 27 03 258 265

[21] Moore D Keat R Does dental appearance impact on employability in adults? A scoping review of quantitative and qualitative evidence Br Dent J 2020(e-pub ahead of print). 10.1038/s41415-020-2025-5 33082523

[22] Almedlej R Aldosary R Barakah R Dental esthetic and the likelihood of finding a job in Saudi Arabia. A cross-sectional study J Family Med Prim Care 2020 9 01 276 281 32110604 10.4103/jfmpc.jfmpc_742_19 PMC7014893

[23] Namal N Can G Vehid S Koksal S Kaypmaz A Dental health status and risk factors for dental caries in adults in Istanbul, Turkey East Mediterr Health J 2008 14 01 110 118 18557458

[24] El-Yousfi S Jones K White S Marshman Z A rapid review of barriers to oral healthcare for people with protected characteristics Br Dent J 2020 228 11 853 858 32541747 10.1038/s41415-020-1637-0

[25] Gao S S Yon M JY Chen K J Duangthip D Lo E CM Chu C H Utilization of a mobile dental vehicle for oral healthcare in rural areas Int J Environ Res Public Health 2019 16 07 1234 30959954 10.3390/ijerph16071234 PMC6480282

[26] AlHobail S Q Baseer M A Ingle N A Assery M K AlSanea J A AlMugeiren O M Evaluation distal caries of the second molars in the presence of third molars among saudi patients J Int Soc Prev Community Dent 2019 9 05 505 512 31620385 10.4103/jispcd.JISPCD_19_19 PMC6792306

[27] Darisi R D Oral diseases and population health: burden and challenges-part-I Oral Health (0974–3960) 2020 16 07 25 28

[28] Sogi G M Khan S A Bathla M Sudan J Oral health status, self-perceived dental needs, and barriers to utilization of dental services among people with psychiatric disorders reporting to a tertiary care center in Haryana Dent Res J (Isfahan) 2020 17 05 360 365 33343844 PMC7737828

[29] Joshi S Suominen A L Knuuttila M Bernabé E Toothbrushing behaviour and periodontal pocketing: an 11-year longitudinal study J Clin Periodontol 2018 45 02 196 203 29178189 10.1111/jcpe.12844

[30] Valadas L AR Fernandes M L Silva M IG Oral manifestations of drug abuse: a review of literature J Young Pharm 2020 12 01 11

